# Current distribution of distributed all-polar cochlear implant stimulation mode measured in-situ

**DOI:** 10.1371/journal.pone.0275961

**Published:** 2022-10-31

**Authors:** Pierre Stahl, Kai Dang, Clair Vandersteen, Nicolas Guevara, Maureen Clerc, Dan Gnansia

**Affiliations:** 1 Department of Research and Technology, Oticon Medical, Vallauris, France; 2 Athena Project Team, INRIA, Université Côte d’Azur, Nice, France; 3 Head and Neck Surgery Institute, Nice University Hospital, Nice Cedex, France; Hannover Medical School: Medizinische Hochschule Hannover, GERMANY

## Abstract

Oticon Medical cochlear implants use a stimulation mode called Distributed All-Polar (DAP) that connects all non-stimulating available intracochlear electrodes and an extracochlear reference electrode. It results in a complex distribution of current that is yet undescribed. The present study aims at providing a first characterization of this current distribution. A Neuro Zti was modified to allow the measurement of current returning to each electrode during a DAP stimulation and was implanted in an ex-vivo human head. Maps of distributed current were then created for different stimulation conditions with different charge levels. Results show that, on average, about 20% of current returns to the extracochlear reference electrode, while the remaining 80% is distributed between intracochlear electrodes. The position of the stimulating electrode changed this ratio, and about 10% more current to the extracochlear return in case of the first 3 basal electrodes than for apical and mid position electrodes was observed. Increasing the charge level led to small but significant change in the ratio, and about 4% more current to the extracochlear return was measured when increasing the charge level from 11.7 to 70 nC. Further research is needed to show if DAP yields better speech understanding than other stimulation modes.

## 1. Introduction

Cochlear implants (CI) are surgically implanted medical prostheses that aim to restore auditory sensations in people with severe-to-profound sensorineural hearing loss and with limited benefit from conventional amplification. CIs bypass most of the peripheral auditory system and electrically stimulate auditory nerve fibres with an array of electrodes placed inside the cochlea. Sound information is transmitted by stimulating each electrode with a charge related to the level of acoustic energy in each frequency band along the cochlea, taking advantage of its tonotopic organization [1]. A large variability in individual hearing performances exist among CI users [2–4]. The causes of this variability are multiple [5] and partly relate to the CI electrical stimulation that affects the electrode-neuron interface. Because the quality of the conveyed spectral information remains critical for speech perception (e.g., in background noise [6]), part of the scientific community focused at investigating the spread of electrical stimulation. The stimulation mode is one factor modifying the electrical spread [7]. Animal data and computer modelling show a continuity of electrical spreading to more central structures (e.g., [8]). To improve this electrode-neuron interface, CI systems provide different stimulation modes for research and/or clinical usage.

The Neuro Zti implant (Oticon Medical, Smørum, Denmark) and its predicate CI system, the Digisonic SP implant, use a stimulation called Distributed All-Polar (DAP). In the DAP stimulation mode, current is injected by one intra-cochlear electrode and returns through all non-stimulating electrodes including an extra-cochlear reference electrode in fractions depending on the impedance of each electrode contact. The goal of the present study is to provide a description of the DAP stimulation mode. It presents first representative data of the current distribution. The effect of amplitude and duration of the stimulation on return currents is also assessed. The implant design does not allow for independent current measurements of the DAP in clinical situation. Therefore, a human cadaver head was chosen as a model.

### 1.1 Stimulation modes

Stimulation driven through the electrodes of a CI system generates electric potentials inside the cochlea, and neural excitation results from the passage of current from one or several stimulating electrodes (described as ‘stimulating terminal’, e.g., intracochlear parallel stimulation on a pair of electrodes) to one or several return electrodes (described as ‘return terminal’, e.g., multiple intracochlear electrodes). Depending on the CI system, the return terminal can be driven by one or several current sources (by providing a pulse in the opposite phase), or by grounding return electrodes.

With an intracochlear stimulating terminal, depending on the return terminal location, CIs can operate according to three types of stimulation modes: Extracochlear (EX, current flows from the cochlea to one or several extracochlear locations), Intracochlear (INT, current remains inside the cochlea), and Combined (COM, current returns are shared inside and outside the cochlea). The stimulation types differ by the role of each of the intracochlear and extracochlear electrodes: they can be used as stimulating terminal, as return terminal, or not used (i.e., in an open state).

**Fig 1:**
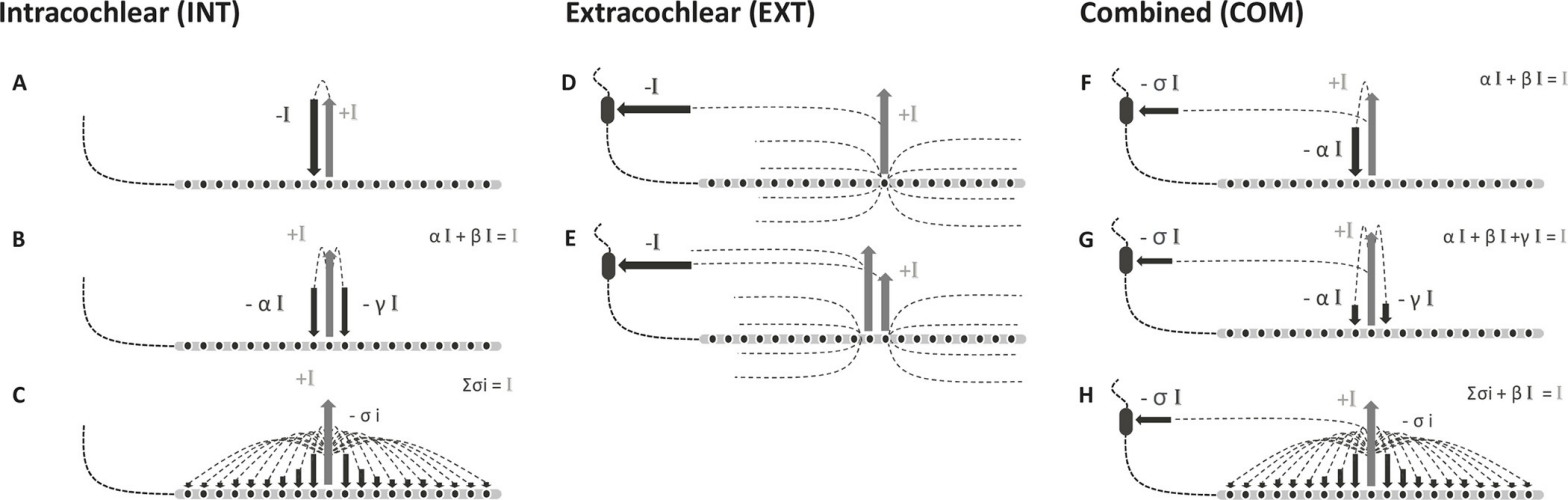
Diagram representing stimulation modes of INT (left), EXT (mid) and COM (left) types, and their expected current paths. **(A)** Bipolar, **(B)** Tripolar and **(C)** Common Ground belongs to the INT type; **(D)** Monopolar and **(E);** Current Steering belong to the EXT type; **(F)** partial-bipolar, **(G)** partial-tripolar and **(H)** Distributed All-Polar to the COM type. The stimulating and returning paths are presented by grey and black arrows, respectively.

In the EX types, the return terminal is one or several extracochlear electrode contacts (e.g., Monopolar mode [2] and Current Steering as described in [9], Fig 1). In Monopolar stimulation, all the current is deliberately flowing out of the cochlea. This mode allows for low current levels to stimulate the auditory nerve fibres [2,3]. Although stimulation modes in currently available devices produce equivalent results averaged across patient population, attempts have been made to improve performance by reducing the current spread. The INT types are intended to reduce current spread compared to EX. They include stimulation modes that aim to contain the current within the cochlea thanks to one or several intracochlear electrodes used as the return terminal while no extracochlear electrode is used. INT stimulation can either be done in a controlled (e.g., Bipolar [10,11], Tripolar [10], and All-polar as described in [12,13]) or uncontrolled manner (e.g., Common-Ground [11]). For instance, Bipolar and Tripolar provide a successively more focused electrical field than Monopolar as shown in animal models [7]. Such high focussing has possible negative consequences for patients with significant neural degeneration. High current levels are required to cover the full dynamic of the patient’s audibility range [14], and these levels may become highly variable [15]. Patient outcomes may not be better than with EXT types [14] while power consumption is increased. The Common-Ground stimulation mode (Fig 1C), an example of INT type, is used since the first decades of multichannel CIs. All non-stimulating intracochlear electrodes are used together as ground to provide the return current path. The uncontrolled current pathway is distributed between all the remaining electrodes and dependent of individual electrode impedances.

The COM type combines both INT and EX types (intra- and extracochlear electrodes simultaneously used as return terminal) and aims to trade-off between current focusing and power consumption. All-polar (as described by [16]) and partial-Tripolar (p-tripolar, [17]) are examples of the COM type. P-tripolar uses two intracochlear and one extracochlear electrode as the return terminal. Attributing different amounts of current returning to the extracochlear electrode could lead to a compromise between Monopolar and Tripolar for spectral resolution and operating range of current levels [18,19]. Arenberg et al. [15,20] showed that actively modifying the ratio of current returning to the extracochlear reference electrode led to systematic changes in the amount of charge required to evoke thresholds. This scaled from low charge when stimulating in Monopolar, to high charges when stimulating in full-Tripolar. Quantification of spectral excitation with psychophysical tuning curves lead to similar observations, with sharper tuning curves as the ratio increased towards Tripolar. Sharper tuning curves with p-Tripolar than with Monopolar configurations could be observed even when stimulation levels for p-Tripolar were increased to match loudness [21], with larger dynamic ranges [14].

### 1.2 Oticon medical neuro CI system stimulation

#### Stimulation mode and associated pulse waveform

Similar to the Common Ground mode, the Distributed All Polar (DAP) stimulation mode uses all electrodes to contribute for the current return. All terminals are turned into a ground state, including an additional extracochlear return electrode. The overall current distribution pattern of DAP makes it comparable to a COM type that uses non-controlled returns. As described by [18], the total return current can be separated into two fractions: the intracochlear return fraction “INT” kept inside the cochlea, and the extracochlear fraction “EXT” flowing out of the cochlea to the reference electrode (see Fig 1G). The ratio between INT and EXT depends on the relative impedances of each electrode contact and will vary between patients and is affected by physiological processes affecting the volume conduction in and outside the cochlea, such as ossification. In this study, we will characterize the INT/EXT ratio in a single human head specimen in different stimulating conditions.

In DAP mode, the return electrodes are not actively participating in the pulse shape. Implant power is required only for generating the first phase (anodic, i.e., active) while the current is returning to all non-stimulating grounded electrodes. In the second phase (cathodic, i.e., non-active), the stimulating electrode with its capacitor in series (nominal value of 220nF ±10%) is connected to ground. The resulting charge recovery follows an exponential decay function.

#### Loudness coding and typical fitting parameters

Loudness coding can be controlled by modulating the pulse amplitude, the pulse duration, and/or the stimulation rate of electrical stimulation [22–26]. Most CIs modulate pulse amplitudes to evoke different loudness levels, while pulse duration is roughly fixed. The Oticon Medical system uses a fixed pulse amplitude while modulating the pulse duration. The amplitude ranges from 222 to 2000μA with a typical value of 444μA. The pulse duration ranges from 10 to 115 μs, with typical threshold and comfort values of 30 and 60 μs, respectively. Typical electrode impedance is 3 kΩ (7 kΩ is the maximum recommended value in the clinical fitting software). The clinical impedance feature involves the measure of the total output voltage required to generate a pulse during a DAP stimulation. No estimation of the sub-interactions between electrodes is assessed.

A characteristic of DAP is that the current freely returns to each grounded contact in an uncontrolled manner. The exact current returning on all electrodes is related to electrode impedances and is yet to be characterised. With the aim to describe the DAP current distribution, measurements were made to quantify the current at each non-stimulating electrode in a single human cadaver head. Measurements were performed at different pulse amplitudes and pulse durations to evaluate the effect of stimulation parameters on return currents.

## 2. Materials and methods

The study was conducted at the Department of Anatomy of the Nice University Hospital, France. The department is approved by the French medical authorities for experiments and for medical training. The experiment did not require any additional ethical approval.

### 2.1 Hardware

**Fig 2:**
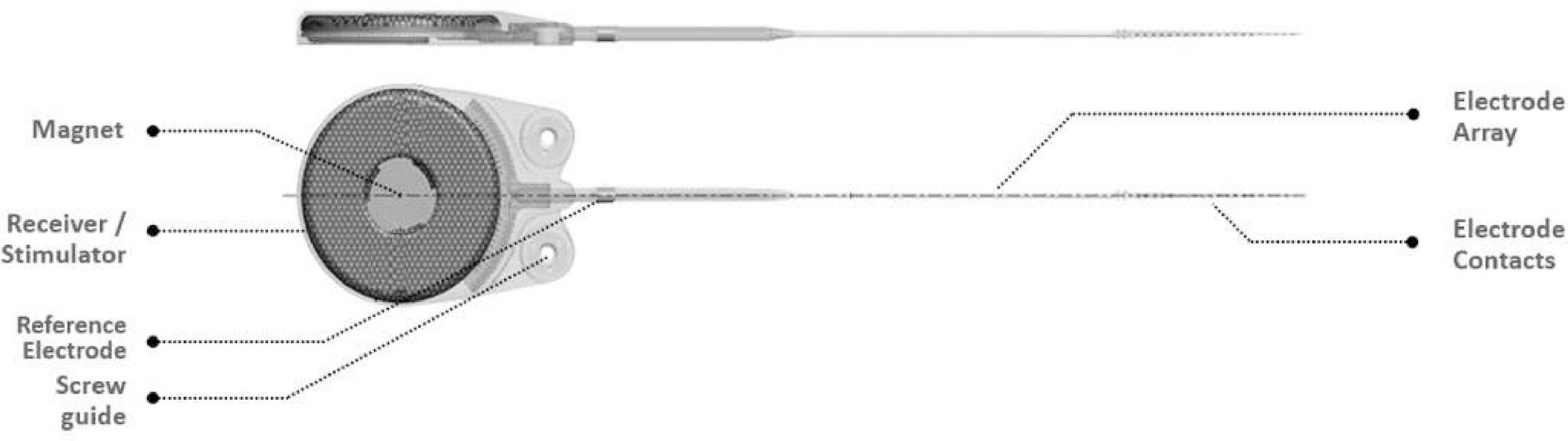
Representation of the Neuro Zti Cochlear implant.

The electrical stimulation was performed by using a standard Neuro Zti cochlear implant receiver / stimulator (Oticon Medical; see [27] with an EVO electrode-array and a Neuro One speech processor [28]. As shown in Fig 2, the Neuro Zti receiver / stimulator lead consists of a single silicone tubing harbouring one large cylindrical extracochlear electrode proximal to the CI stimulator attached to an electrode array with 20 smaller intracochlear cylindrical electrodes. The distance separating the extracochlear electrode and first intracochlear electrode is 76.4 mm. The extracochlear reference electrode has a diameter of 2.22 mm and a length of 2.5 mm for a surface area of 17.4 mm^2^. The intracochlear electrode contacts are 0.47 mm long and are spaced by 0.7 mm. In the EVO electrode array the electrode diameter varies between the proximal and distal halves of the array. The array and electrode diameter are 0.5 mm for the 10 proximal electrodes (basal section of the array) and 0.4 mm for the 10 distal electrodes (apical section of the array). The surface area of the reference electrode is therefore approximately 25 times larger than the average surface area of the intracochlear electrodes.

In lieu of clinical fitting software, the CI stimulator was controlled via custom software for the present study. The custom software allowed to adjust automatically both pulse amplitude and pulse duration. The pulse amplitude varied from 0.222 to 2 mA with 0.022 mA step size. The pulse duration varied from 10 μs to 115 μs with a 1 μs step size. The experiment used a total of six stimulation pulse waveforms combining different amplitude and duration values.

In the Neuro CI system, a current source drives the stimulation to deliver the anodic phase of the pulse. The subsequent cathodic phase is ensured by direct current blocking capacitors inserted in series with each electrode to allow charge balancing following an exponential decay function. The extracochlear reference electrode is connected directly to the ground. Thus, the capacitor voltage values were used to measure the returning current at each of electrode contacts that are grounded during DAP stimulation.

### 2.2 In situ measurement setup and signal acquisition

#### Implantation procedure

An experienced ENT surgeon implanted the left ear of an ex-vivo human head with a standard surgical procedure, i.e., mastoidectomy and posterior tympanotomy with round window electrode insertion approach. In this case the electrode array could not be fully inserted, and the surgeon visually confirmed that the four most basal contacts were out of the cochlea (E1 to E4), with the fourth being at the round window. The cochlea was filled with physiological saline solution but not sealed after electrode insertion. All visible electrodes (reference electrode and the four out of the cochlea) were confirmed to be in contact with the tissue. The skin flap was closed. Electrode impedances were checked to be in the normal operating range (i.e., 2.5–3.5 kΩ) but not stored. The four extracochlear contacts were considered similarly to all other intracochlear contacts. No specific control was applied on the head’s temperature, although the room was at a constant temperature of 20°C.

#### Measurement setup

**Fig 3:**
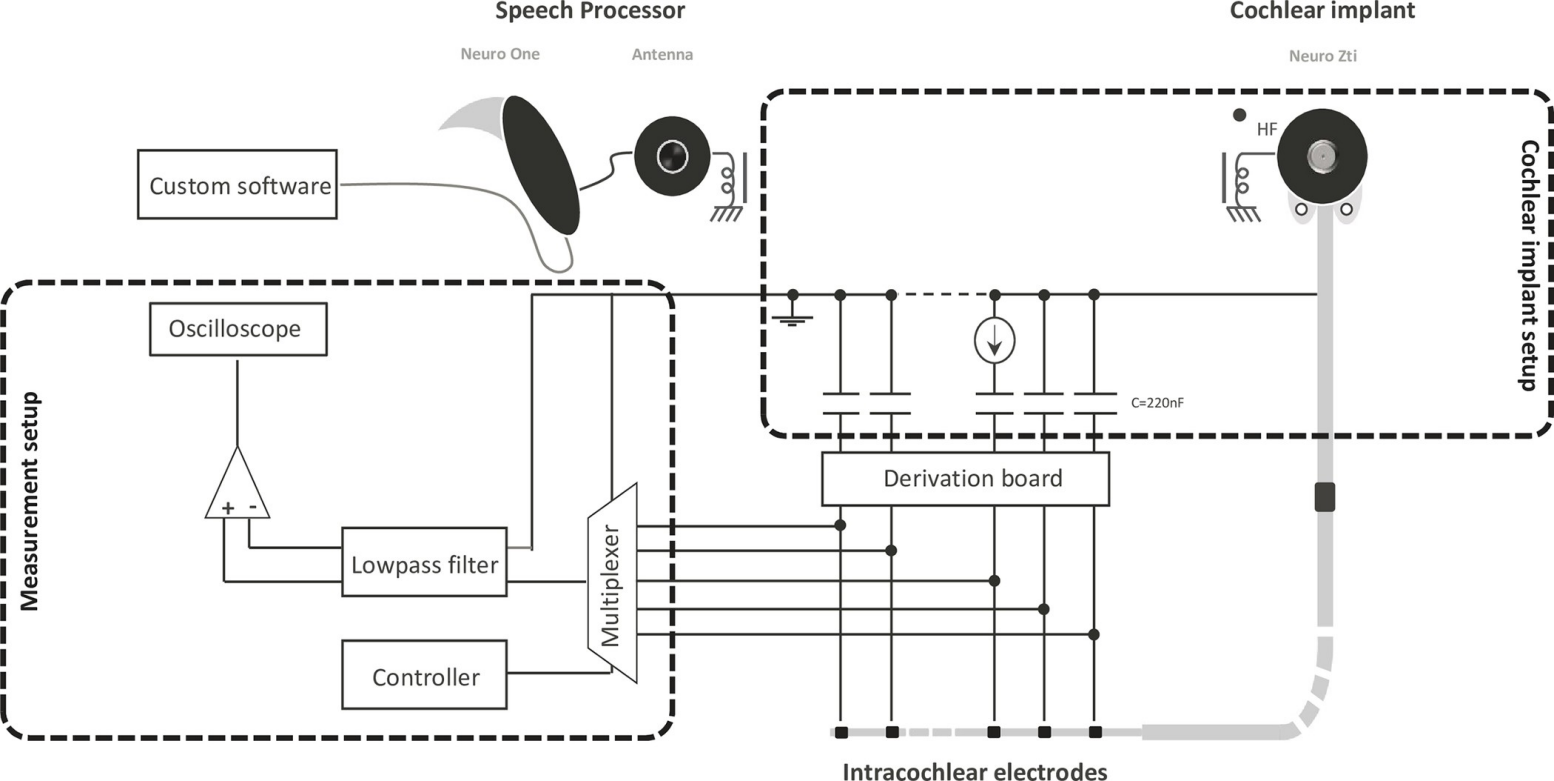
Schema representing the implant and the measuring system.

The Neuro Zti implant has 20 electrodes corresponding to 20 stimulation channels. To reconstruct a complete map of current distribution for each possible stimulating electrode, the current must be measured individually at each of the remaining 19 return electrodes. For each stimulating electrode, these measurements were performed on each return electrode in a serial manner. For that purpose, an in-house analog multiplexer was designed to automate and speed up the switching and recording process. The Neuro Zti cochlear implant was modified so that a board was inserted between the CI stimulator and the electrode array. This board allowed the connection with the multiplexer. Fig 3 shows a schema of the multiplexer attached to the Neuro Zti implant. The device had three multiplexer chips (16 channel analog multiplexers, 74HC4067, Nexperia) that allow for 48 input channels for switching. The ON resistance of the selected channel *R*_*ON*_ is 80 Ω, with 8 Ω mismatch between channels. A micro-controller (Arduino UNO) was used to drive the selection of the input channel according to the PC commands. The multiplexer allowed the automatic switching of the recording between different electrodes on the array. The voltage *V*_*b*_ on each blocking capacitor was then recorded to estimate the current *I*_*b*_ of the corresponding intracochlear electrode, using Eq 1, where *C*_*b*_ is the size of the blocking capacitor, in farad:

$$I_{b}=C_{b}-\frac{{dV}_{b}}{dt}$$ (1)

The current through the extracochlear reference electrode *I*_*ref*_ is computed by the Eu 2, where *I*_*s*_ is the stimulation current, **N** is the set of non-stimulating intracochlear electrodes and *I*_*n*_ is the current on the corresponding electrode *n*:

$$I_{Ref}=I_{S}-\sum I_{n},\;n\in N$$ (2)

To remove any possible noise of the recording, an analog low-pass filter was inserted before the differential probe to reduce noise levels so that the oscilloscope could track and record the correct stimulation waveform. The low-pass filter was a 3-order Butterworth filter with a 1 *MHz* cut-off frequency.

#### Signal acquisition

The voltage signal was stored and analysed with a PicoScope 2205A digital oscilloscope with TA045 differential probe (Pico Technology Ltd., Cambridgeshire, UK). The differential probe had a 500 kΩ input resistance and a 7 pF input capacitance. The signal was recorded with 12 bits amplitude resolution and 4 MHz sampling frequency. A PC with in-house software controlled the communication and synchronization between the stimulation and acquisition. The post processing of the acquired signal, including denoising, waveform extraction and amplitude measurement, was completed using MATLAB (MathWorks Inc., Natick, MA, USA).

### 2.3 Stimulating conditions

**Fig 4:**
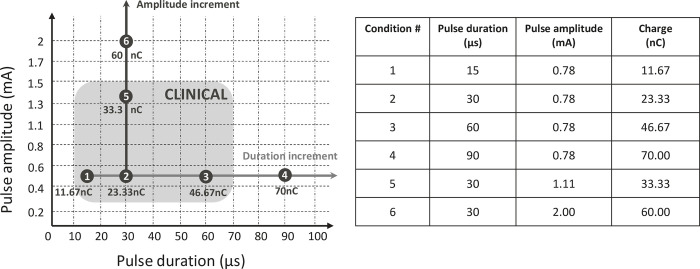
(**A)** Distribution of the stimulation parameters across conditions. The conditions 1, 2, 3, and 5 were defined to be within the range of charge levels observed in routine clinical practice conditions [27]. The conditions 4 and 6 represented extreme stimulation charge levels (close to max system output, not observed in clinical practice) by increasing either stimulus amplitude or duration. (**B)** Table providing full description of the pulse characteristics used for all conditions.

Six different stimulating conditions combined different pulse durations and amplitudes. When pulse durations were modified (cf. Fig 4A., Stim conditions # 1, 2, 3, and 4), they ranged from 15 μs to 90 μs while amplitude was kept fixed at 0.78 mA. When pulse amplitudes were modified (cf. Fig 4B. 1, Stim conditions # 2, 5, and 6), they ranged from 0.78 to 2 mA and pulse lengths were kept fixed at 30 μs.

For each of the 6 stimulating conditions and 20 stimulating electrodes, a scan consisting of 20 oscilloscope recordings was performed from the base to the apex of the array (i.e., from electrodes 1 to 20). The oscilloscope measured voltage over time between each electrode and the reference electrode during the stimulation, which was converted into current following Eq 1. Since the extracochlear electrode has no series capacitor, it was not possible to connect this contact to the oscilloscope and measure its associated voltage. The 20 scans were repeated 3 times to eliminate noise for each of the 120 possible stimuli, resulting in a total of 7,200 oscilloscope measurements.

## 3. Results

### Oscilloscope waveform recordings

**Fig 5:**
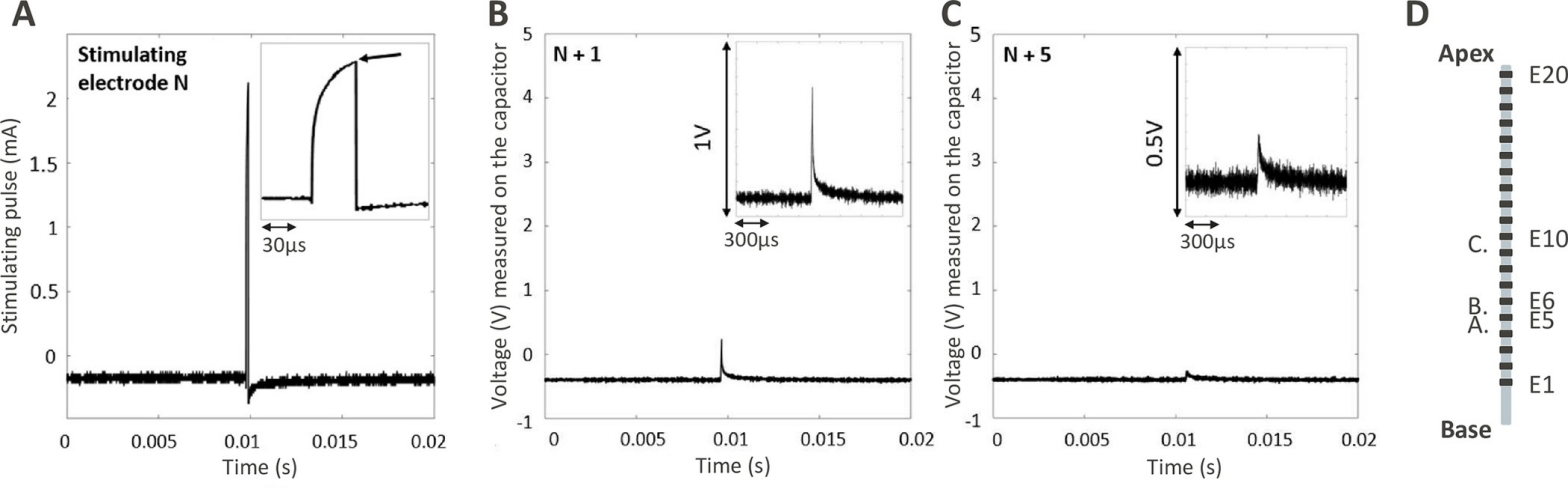
**A, B, C.** Oscilloscope recordings for electrodes E5 (stimulating), E6 and E10 (recording), a zoom of each recording is provided in the top-right small panels. (**A)** Oscilloscope view showing the pulse shape on the stimulating electrode (N, electrode E5), by converting the recorded voltage in current with the averaged clinical impedance of 3 kΩ. The arrow shows the measurement point on the oscilloscope trace, i.e., at the anodic phase offset, where the peak is maximum. (**B)** Oscilloscope view showing the raw voltage recorded on an adjacent returning electrode’s capacitor (N+1, E6). (**C)** Oscilloscope view showing voltage recorded on another adjacent returning electrode’s capacitor (N+5, E10). (**D)** Mock diagram of the electrode array and the respective places of electrode E5 (A), E6 (B) and E10 (C). The stimulating electrode was always E5.

Fig 5 provides an example of oscilloscope recording traces for three electrodes (electrodes 5, 6 and 10 referred as E5, E6 and E10; where E1 is the most basal electrode and E20 the most apical, as shown in Fig 5D). Fig 5A shows the pulse shape from a recording on the stimulating electrode capacitor. The resulting voltage was converted in current to show the pulse shape, using the typical clinical impedance of 3 kΩ. The full pulse is depicted including the anodic-first active phase and the cathodic phase. The non-rectangular shape of the anodic phase is due to the capacitive effect of the physiological saline solution. The other panels from Fig 5B and 5C show unprocessed voltage oscilloscope recordings of the return electrodes, depicting the loading and unloading voltages of the return electrodes capacitors that are typical of the anodic and cathodic phases. The high and fast voltage increase represents the effect of the anodic phase, during which the current is quickly spreading from the activated electrode to all return electrodes. The slower exponential decrease represents the cathodic phase. The recorded voltage decreased as the distance between the stimulating and measured electrode increased (e.g., N+1 vs. N+5).

### Maps of current distribution

**Fig 6:**
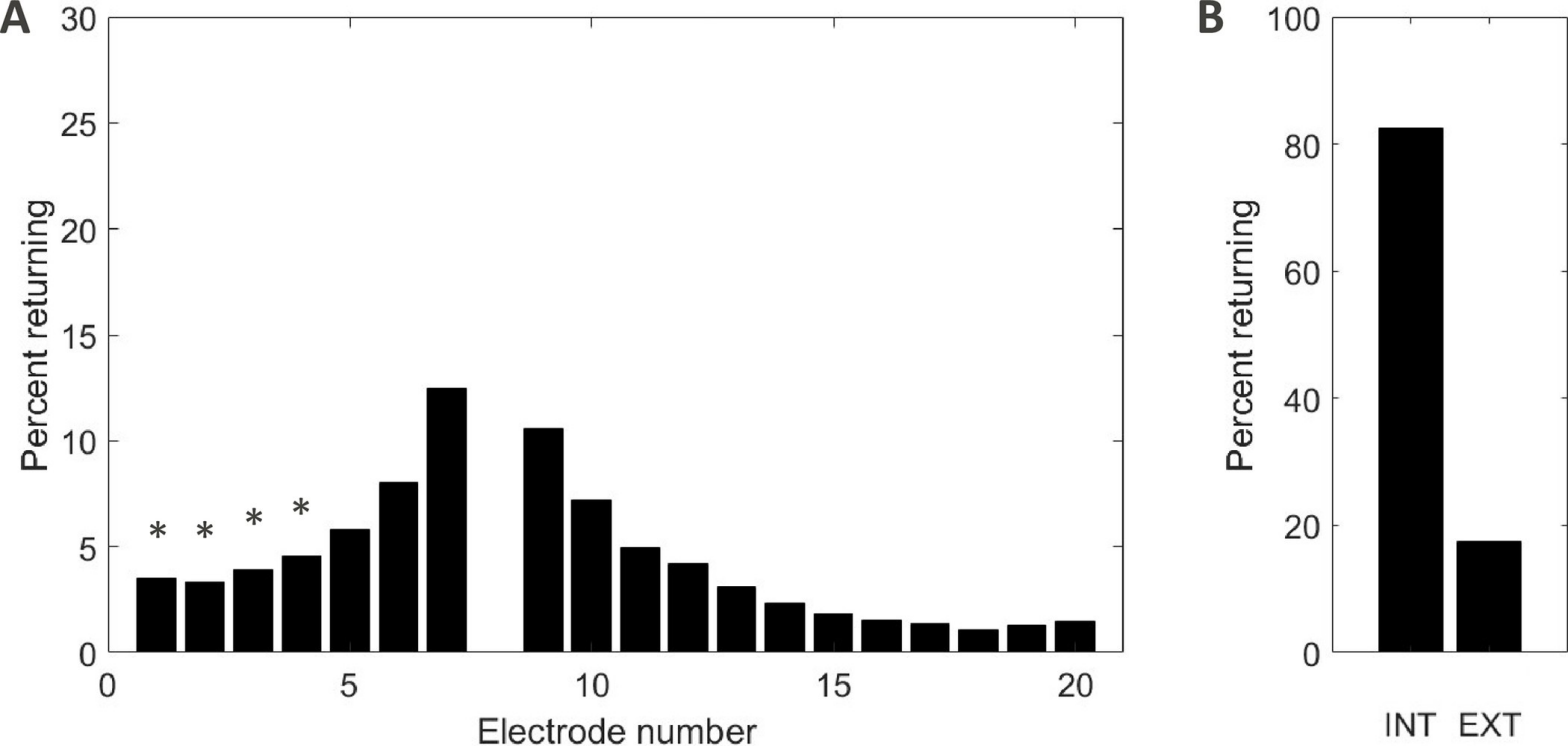
(**A)** Example of current returning distributions on the Intracochlear electrodes taken from the stimulating condition 3, electrode 8. The stimulating electrode, E8, is generating 100% of current and is not depicted in the diagram. (**B)** Resulting INT estimation (calculated from the sum of all intracochlear currents) and EXT (inferred from 100 minus INT). The symbols (*) show the electrodes out of the cochlea.

For every stimulating electrode, INT was calculated to build representative maps of the entire array and estimate EXT. It was calculated as follows. First, a map distribution of current was built for each stimulating electrode and condition. It was then expressed in percent of the stimulating current. An example of such resulting map is given in Fig 6A. Then, and for this map, all percent returning current coming from non-stimulating electrodes (VGrounded in Eq 3) were summed to estimate INT. The INT and EXT are represented in Fig 6B.


$$\int \left(\%\right)=\sum V_{Grounded}(\%)=\frac{100}{N}(N_{+1}+N_{+2}\ldots +N_{+19})$$ (3)

This was used to infer EXT (Eq 4), which is the difference between the total current (i.e., 100%) and INT.


EXT(%)=100−∫(%) (4)

**Fig 7:**
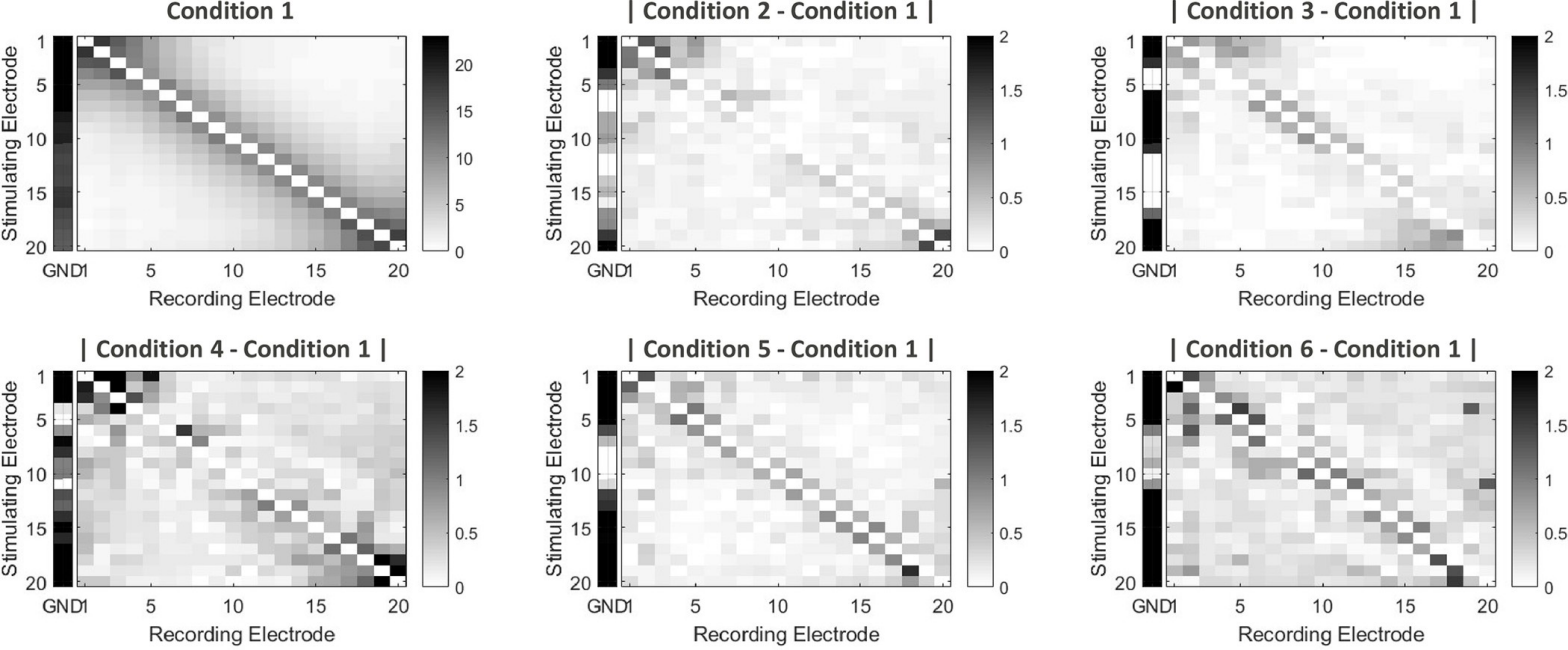
Matrixes of current distribution from the different stimulating conditions. The top left matrixes color-code shows percent of the generated current returning to each INT electrode (from 1 to 20), and their calculated returning to the reference EXT electrode (GND) for the stimulating condition 1. The other panels show the differences in current distribution between the stimulating conditions 2 to 6 and the stimulating condition 1.

Fig 7 shows an overall picture of the current distributions and their differences between stimulating conditions. The top left panel shows the grounding distribution matrix for the condition 1 where the charge level used for the stimulation is the lowest (11.7nC). The empty diagonal represents the stimulating electrode where the current was generated, while the darker shades indicate that an increasing percentage of the injected charge is returning to this specific contact. The other panels represent the absolute difference between the conditions 2–6 and the baseline condition 1. White areas indicate no difference while darker areas show a difference in current returning on this contact. The differences in current returns when increasing the charge level is small (mainly lower than 2%) and located at the contacts closest from the stimulating electrode, more prominent for the extreme apical or basal stimulating electrodes. The condition 4, where the charge level used for stimulation was the highest (70nC), showed the strongest differences.

### INT and EXT in the Distributed All-Polar stimulation mode

**Fig 8:**
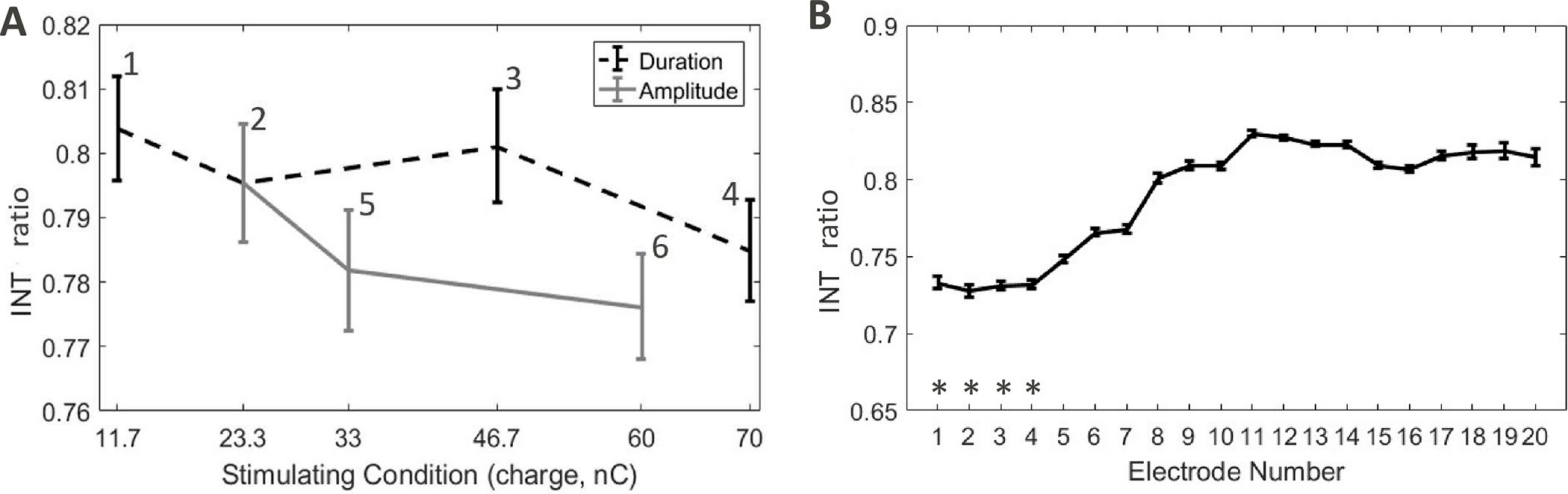
Distributions of INT. (**A)** Between stimulation condition, therefore averaged across electrodes. (**B)** Between electrodes, therefore averaged across repetitions and conditions. Error bars show standard error. Labels overlayed on the functions represents the stimulating conditions. The symbols (*) show the electrodes out of the cochlea.

The effect of stimulating condition and stimulating electrode on the recorded INT was investigated through a two-way analysis of variance (ANOVA). Before analysis and to keep the proportionality of the data, an arcsine square root transformation was performed. Both stimulating condition [F(1,5) = 48.3, p<0.001] and stimulating electrode [F(1,19) = 160.4, p<0.001] showed statistically significant effect, meaning that the stimulating condition and the stimulating electrode changed INT and EXT. Although statistically significant, effect sizes were moderate at best for the stimulating condition, where the largest difference in the INT/EXT ratio was around 4%. Within the range of clinical conditions (i.e., conditions 2, 3, and 5), the largest difference in the INT/EXT ratio was 2% (Fig 8B). Modifying the stimulating electrode showed a larger effect with INT/EXT ratio differing more than 10% between apical and basal electrodes. Fig 8 summarizes the findings by showing the independent effect of the stimulating condition and of the stimulating electrode on INT.

### Current distribution of Distributed All-Polar

**Fig 9:**
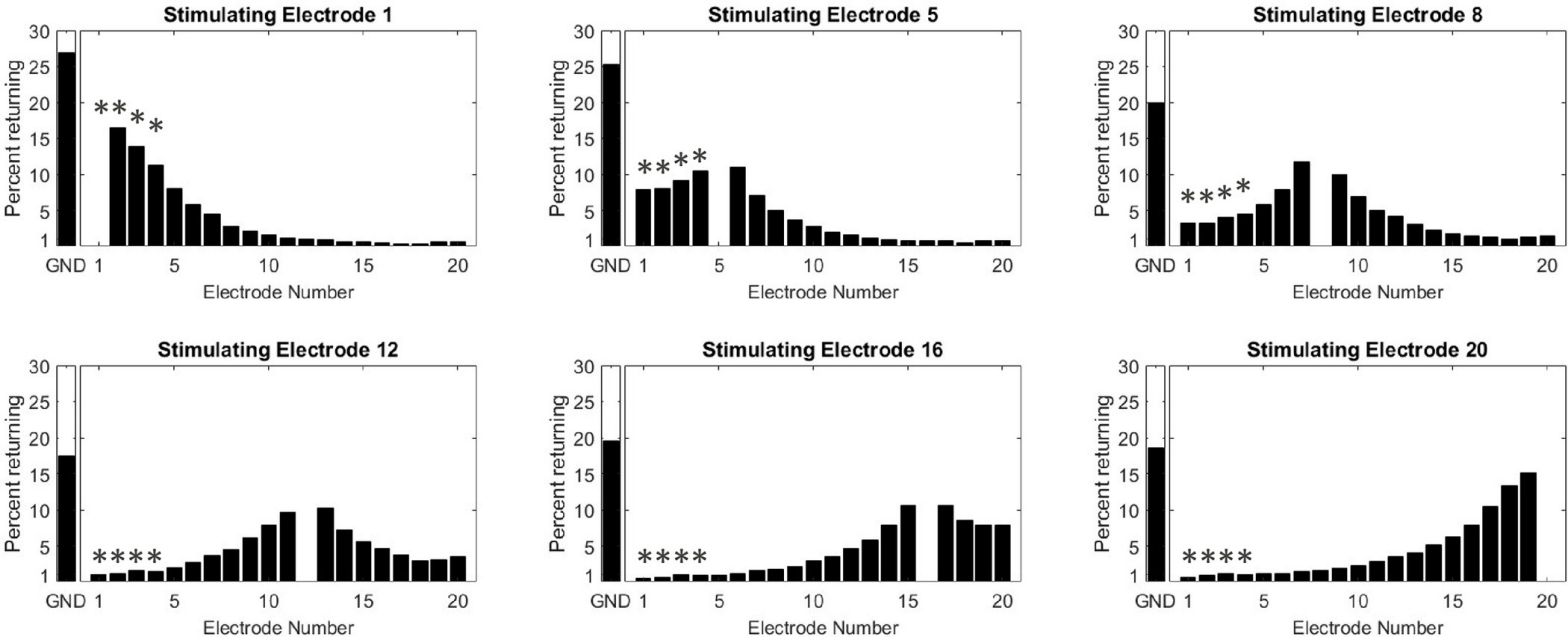
Example of returning current distributions from data pooled among stimulating conditions along the electrode array and for 6 different stimulating electrodes; E1, E4, E8, E12, E15, and E20. Left panels of each maps represent the percent returning to the ground electrode, i.e., the EXT. Right panels represent the percent returning on each intracochlear electrodes (and if summed, the INT). The symbol (*) shows the electrodes out of the cochlea.

Finally, all available data were pooled to estimate the overall current distribution with DAP stimulation. To estimate the overall current distribution independently from the stimulating condition, the percentage of current returning to each electrode was averaged across repetitions and conditions to create a single matrix representing the returning current distribution for all 20 stimulating electrodes. Six transversal slices of the matrix are represented in Fig 9, showing the map returning currents along the electrode array, when moving from stimulating electrode E1 to E20. INT can be inferred from the sum of all intracochlear currents (i.e., summation from E1 to E20). EXT is the percentage of current returning to the extracochlear ground electrode (noted GND).

## 4. Discussion

### Anatomical model

This study aimed to present representative data of the current distribution resulting from DAP. It was assessed by measuring the voltage across the implant’s blocking capacitors that load/unload during a DAP stimulation. The implant design does not allow for such measurements in clinical situation (i.e., in healthy CI patients), although it would have provided more samples for the tests and realistic conditions. In lieu of living patients, an ex-vivo human head was used. This was chosen to obtain relevant measures of current distribution over other models for several reasons. Although conduction models are interesting [29], they are commonly tuned or validated by real current measures. Since all electrodes are contributing to create the returning pattern of DAP, it was unclear whether current computational models could faithfully recreate such a complex and previously uncharacterised pattern. Second, even though most cochlear structures and surrounding elements can be identified via CT scan or MRI section images [30,31], recreating a relevant physical cochlear model for testing remains technically challenging.

The present study has three major limitations. First, electrode array insertion was incomplete, with the four basal electrodes outside the cochlea, which corresponds to an insertion depth of 20.5 mm. In comparison, clinical insertions depth for this array ranges from 23 to 25 mm [32,33]. Second, measurements were conducted directly after insertion and without any tissue growth near the cochlear. This differs from a standard clinical in-vivo situation where fibrotic tissue occurs post-surgically. Therefore, differences in current distribution between the present study and a clinical situation could occur. Third, additional data from other head models could strengthen the dataset and conclusions, as cochlear anatomy and electrode array position could affect return currents. The present data still provide a preliminary estimation of the current distribution with the DAP stimulation mode, whilst keeping the limitations of the study in mind.

### Effect of the stimulating electrode

**Fig 10:**
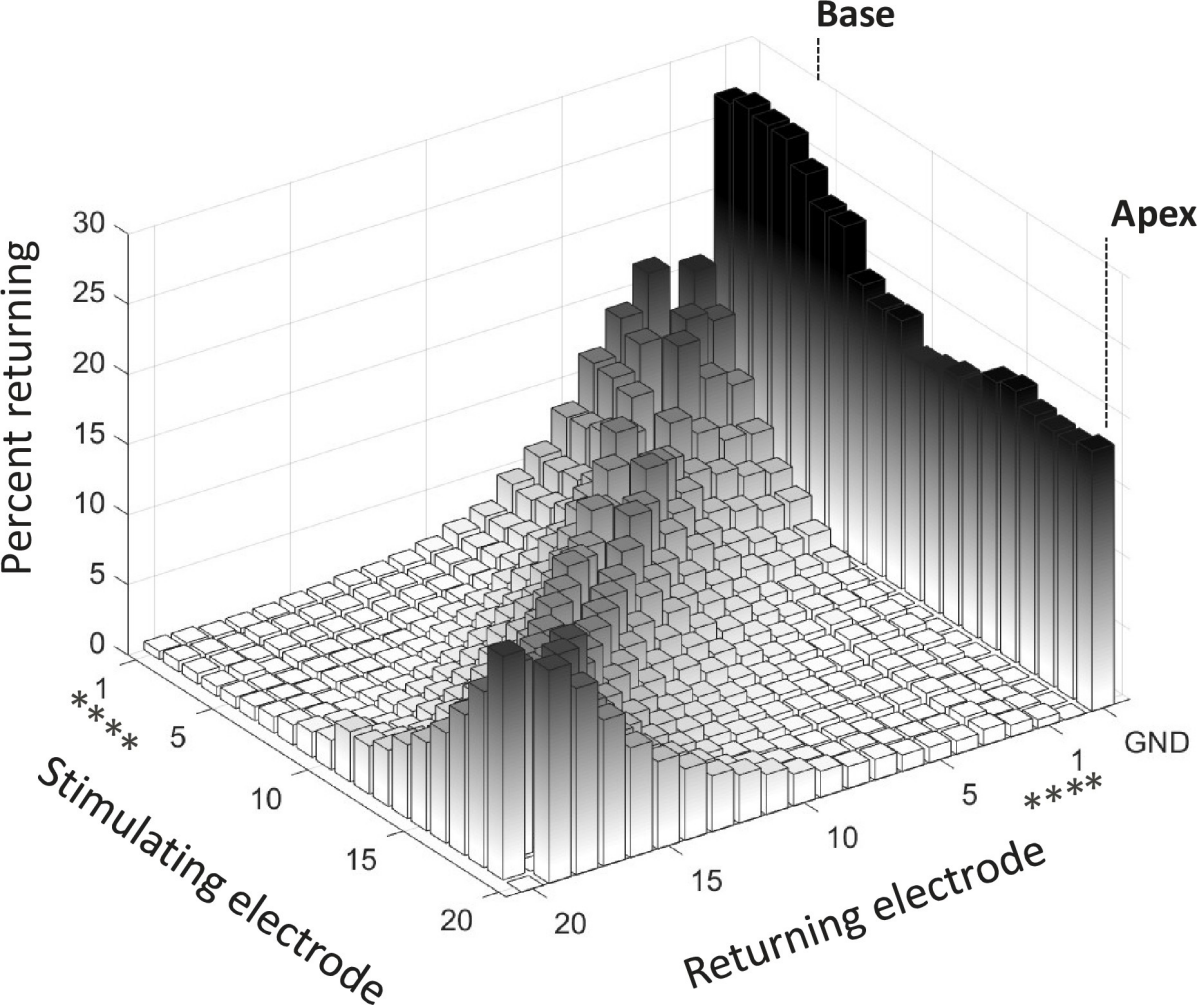
Pattern of DAP stimulation. Current distribution in ex-vivo measurement representing the intra- and extracochlear current returning to each electrode contact. The returning current is expressed as a percentage of current generated by the corresponding stimulating electrode. The double arrows are pointing to the electrodes 1–4, visually detected to be out of the cochlea. The symbol (*) shows the electrodes out of the cochlea.

INT differed per stimulating electrode position and shows an increase in INT of 10% from the most basal stimulating electrode to the most apical. The four extracochlear electrodes (E1 –E4) showed the lowest INT of around 73%. It then gradually increases to about 83% in the basal cochlear turn (up to E11) then stabilises to this value (Fig 8 showing INT, Fig 10 showing EXT). The results may differ from a clinical situation since the cochlear opening was not sealed in this experiment.

### Effect of increasing pulse amplitude or duration

Many stimulation strategies aim for high-rate non-simultaneous stimulation and use biphasic pulses of a short duration of each phase to make this possible. An often-occurring problem for these strategies is that the current source can reach its compliance limit if the electrode impedance is high. DAP allows for a low pulse rate providing the possibility to use long duration low-amplitude pulses, decreasing the chance of hitting the compliance limit. The study also evaluated the effects of pulse amplitude and duration on DAP current returns (see Figs 7 and 8A). Although statistical analysis showed a statistically significant difference between current distributions as a function of amplitude and duration, the effect was rather small. Amplitude modulation showed lower INT than duration modulation at the same range of charge levels, in the magnitude of 2–10%. This effect is not yet understood and should be further evaluated.

### General returning distribution

Fig 10 represents the DAP current distribution as a function of the 20 stimulating electrodes from the most basal (E1) to the most apical (E20). Among all stimulating conditions and stimulating electrodes, the average percentage of EXT current was approximately one fifth of the total current generated (20% EXT, 80% INT). These results confirm that the DAP stimulation mode can be described as a combination of the Monopolar and Common Ground stimulation modes. Two distribution patterns could be observed depending on the position of the stimulating electrode. For both the apical and extra-cochlear basal electrodes, the distribution is asymmetric and more confined to directly neighbouring electrodes. For mid electrodes, only a subset of intracochlear returns accounts for a majority of the total returning current, the EXT and INT N±3 closest electrodes result in 65–70% of the total current. Implications of these two distributions schemes on the patient fitting maps are still unclear. For example, the limited longitudinal current spread observed for apical electrodes may favour higher electrical charge required to reach comfortable sound levels compared to medial electrodes (from the presumed increase in spread of excitation). However, clinical fitting maps of Neuro Zti CI patients show the opposite trend. An example of characteristic fitting map for this device can be find in [33].

Current distribution asymmetries at the extreme apical (E17, E18, E19, E20) and basal (E1, E2, E3, E4) electrodes may also lead to abnormal pitch sensation (i.e., confusions or reversals in pitch). Because the current returning is not evenly distributed between the sides of the stimulating electrode, the resulting centre of excitation may be shifted to more medial regions. Abnormal pitch sensations were already observed in the Common Ground mode [11] and DAP may also suffer from the same discrepancy, although the origin of pitch reversals with the Common Ground remains unclear and may occur for a different reason (e.g., if impedances are very irregular across the array). In DAP, the addition of a Monopolar stimulation from an extracochlear return is assumed to partially mitigate this issue, but this is yet to be evidenced. Further research on pitch ranking with the DAP stimulation mode is required.

DAP has been used for over two decades, in the Digisonic SP (e.g., [34,35] and the Neuro CI systems [27,28]). As of today, it remains the only stimulation mode that belongs to the COM type and proposed by default in clinically available CI systems. It is not shown to offer better performances in speech recognition than other devices [28,36]. However, a rising interest using DAP that shows to minimize cases of facial nerve stimulation (FNS) still occurring in about 5.6% of the CI population [37]. For instance, two studies evaluated re-implantations of patients issuing FNS with a CI system, replaced by a Neuro Zti CI and ended with a complete resolution of the problem [33,38]. The main contributor for this FNS occurrence reduction is still unclear and may be related to the stimulation mode, the pulse waveform, or another parameter of the electrical stimulation. Future psychophysical and real-life experiment are required to better identify the clinical effects of DAP.
